# Association Between Self-Reported Protective Behavior and Heat-Associated Health Complaints Among Patients With Chronic Diseases in Primary Care: Results of the CLIMATE Pilot Cohort Study

**DOI:** 10.2196/58711

**Published:** 2024-11-04

**Authors:** Arne Jordan, Julia Nothacker, Valentina Paucke, Klaus Heinz Hager, Susann Hueber, Arian Karimzadeh, Thomas Kötter, Christin Löffler, Beate Sigrid Müller, Daniel Tajdar, Dagmar Lühmann, Martin Scherer, Ingmar Schäfer

**Affiliations:** 1 Institute and Outpatients Clinic of General Practice/Primary Care University Medical Center Hamburg-Eppendorf Hamburg Germany; 2 Institute of General Practice and Palliative Care Hannover Medical School Hannover Germany; 3 Institute of General Practice University Hospital Erlangen Friedrich-Alexander-Universität Erlangen-Nürnberg Erlangen Germany; 4 Institute of Family Medicine and General Practice University Hospital Bonn Bonn Germany; 5 Institute of Family Medicine University Medical Centre Schleswig-Holstein, Campus Lübeck Lübeck Germany; 6 Institute of General Practice Rostock University Medical Center Rostock Germany; 7 Institute of General Practice University of Cologne Cologne Germany

**Keywords:** climate change, online survey, open internet data, climate, environment, rising temperature, heatexposure, chronic disease management, epidemiology

## Abstract

**Background:**

As a result of climate change, exposure to high temperatures is becoming more common, even in countries with temperate climates. For patients with chronic diseases, heat poses significant health risks. Empowering patients is a crucial element in protecting the population from the adverse effects of heat. In this context, self-reports of protective behavior are often used to gain a mutual understanding of patients’ issues. However, the extent to which self-reported behavior is associated with health complaints remains unclear.

**Objective:**

This study aims to describe the association between light to moderate heat and health complaints in everyday life, and to analyze whether self-reported protective behavior and related psychosocial factors are linked to these complaints.

**Methods:**

We conducted a pilot cohort study using internet climate data merged with an online survey of patients with chronic diseases recruited through general practitioner practices. Patients were eligible if they were 18 years or older and had at least one chronic disease. The heat was modeled using temperature and humidity data. Health complaints were assessed through up to 7 follow-up evaluations on the hottest day of each week during the observation period. Data were analyzed using 3 nested models with mixed effects multivariable linear regression, adjusting for random effects at the climate measuring station and participant levels. Model 1 included heat exposure, sociodemographic data, and chronic diseases. Model 2 added protective behavior and health literacy, while model 3 incorporated self-efficacy and somatosensory amplification (ie, the tendency to catastrophize normal bodily sensations such as insect bites).

**Results:**

Of the 291 eligible patients, 61 (21.0%) participated in the study, providing 294 observations. On average, participants were 61 (SD 14) years old, and 31 (51%) were men. The most prevalent conditions were cardiovascular diseases (n=23, 38%) and diabetes mellitus (n=20, 33%). The most commonly reported symptoms were tiredness/fatigue (232/294 observations, 78.9%) and shortness of breath (142/294 observations, 48.3%). Compared with temperatures of 27°C or lower, a heat index between over 27°C and 32°C (β=1.02, 95% CI 0.08-1.96, *P*=.03) and over 32°C (β=1.35, 95% CI 0.35-2.35, *P*=.008) were associated with a higher symptom burden. Lower health literacy (β=–0.25, 95% CI –0.49 to –0.01, P=.04) and better self-reported protective behavior (β=0.65, 95% CI 0.29-1.00, *P*<.001) were also linked to increased symptom burden but lost statistical significance in model 3. Instead, lower self-efficacy (β=–0.39, 95% CI –0.54 to –0.23, *P*<.001) and higher somatosensory amplification (β=0.18, 95% CI 0.07-0.28, *P*=.001) were associated with a higher symptom burden.

**Conclusions:**

Compared with colder weather, light and moderate heat were associated with more severe health complaints. Symptom burden was lower in participants with higher self-efficacy and less somatosensory amplification. Self-reported protective behavior was not linked to a lower symptom burden. Instead, we found that patients who tended to catastrophize normal bodily sensations reported both better protective behavior and a higher symptom burden simultaneously.

**Trial Registration:**

ClinicalTrials.gov NCT05961163; https://clinicaltrials.gov/ct2/show/NCT05961163

## Introduction

As a result of climate change, exposure to high temperatures in daily life is becoming more common, even for populations in countries with temperate climates [1]. For example, in 2023, Germany experienced its second sunniest June on record, and it was the fourteenth consecutive June that was warmer than average [2]. For people with chronic diseases, heat poses significant health risks. For example, heat increases the risk of cardiac ischemia and infarction, while dehydration due to heat can lead to kidney fibrosis and chronic kidney disease [3]. A meta-analysis indicated that a 1°C increase in mean temperature was associated with a 2.1% rise in overall cardiovascular mortality [4]. Similar risks are observed in several other chronic diseases, such as diabetes mellitus [5,6]. In 2023, an estimated 3200 heat-related deaths occurred across Germany [7].

Empowering individuals with chronic diseases to recognize health risks associated with heat and respond appropriately in such situations is essential for protecting the population from the adverse effects of heat. For example, personal cooling strategies can significantly reduce physiological heat exposure and the associated health risks [8]. Currently, a wide range of recommendations from various sources on how to manage heat is available [9-11]. However, the potential health benefits of these recommendations have not been sufficiently researched in everyday conditions, particularly for people with chronic diseases [8].

Helping individuals acquire the knowledge and skills to protect their health is primarily the responsibility of health care system employees, who often receive a high level of trust and are typically competent to communicate sensitive and complex issues [12]. Empowerment can be facilitated, for example, through climate-sensitive health counseling. The primary objectives of such services are protecting and promoting health, as well as imparting knowledge about climate change and its related health risks. These consultations are typically based on patient-centered communication [12].

To develop a mutual understanding of how patients manage heat-related health problems, self-reports of protective behavior against heat are often an essential element of climate-sensitive health counseling. However, it remains unclear to which extent self-reported protective behavior indicates effective protection against the adverse effects of heat. For example, in a recent study from Germany [13], the respondents had gaps in knowledge regarding protective behaviors, and existing knowledge of health risks associated with heat was not consistent with their health behavior. Moreover, in everyday life, simple cooling strategies, such as wearing clothing dampened with cool water [14], were used by only a few people [13].

In late summer 2023, a study was conducted to examine the association between heat, heat-related health complaints, and self-reported protective behavior. However, contrary to expectations, the observation period was unusually cold and rainy, with only light to moderate heat observed. For instance, in Hamburg, Germany, the average temperature between July 16 and August 15, 2023, was below 17°C. As a result, participant recruitment was halted after 10% (61/600) of the intended sample size had been reached, and the collected data were used to pilot the study design, with plans to repeat the study under more favorable conditions in 2024.

The aims of the pilot study were (1) to describe the association between light to moderate heat and health complaints in individuals with chronic diseases in everyday life, and (2) to analyze whether the individuals’ self-reported protective behavior and related psychosocial factors were associated with these health complaints. We hypothesized that (1) light to moderate heat would be associated with greater severity of health complaints, and (2) self-reported protective behavior would be associated with lower severity of health complaints.

## Methods

### Study Design and Population

The CLIMATE (Chronical Illness-Related Limitations of the Ability to Cope With Rising Temperatures) study is a prospective observational cohort study. The CLIMATE study was based on publicly available internet data on temperature and humidity, merged with a voluntary online survey of patients with chronic diseases. The survey was not open to the public but was conducted for a sample recruited by cooperating general practitioner (GP) practices in different regions of Germany. In each practice, personnel were instructed to select 30 eligible patients. Patients were included if they were 18 years or older and suffered from at least one of the following conditions: coronary heart disease or myocardial infarction; heart failure (New York Heart Association stage II or higher); cardiac arrhythmias (treated with medication); peripheral artery disease (Fontaine-Ratschow stage II or higher); stroke or transient ischemic attack; diabetes mellitus type 1; diabetes mellitus type 2 treated with oral medication or insulin; chronic obstructive pulmonary disease (Global Initiative for Chronic Obstructive Lung Disease stage 2 or higher); asthma (Global Initiative for Asthma stage II or higher); renal insufficiency (Kidney Disease Improving Global Outcomes stage III or higher); depressive disorder (treated with medication); anxiety disorders (treated with medication); schizophrenia (treated with medication); and peripheral nervous system diseases (with regularly or persistently occurring symptoms).

Patients were excluded if they lacked the capacity to consent, had severe visual impairment, had insufficient German language skills, or were unable to use an internet browser (eg, due to lack of hardware). The selected patients received a flyer that included patient information, as well as a QR code and a short link to facilitate registration for the online survey. The flyer provided information about the purpose of the study, the expected duration of the survey, the types of data collected, how and where the data would be stored, and the identity of the investigator. More extensive information, including measures for data protection, was provided online before obtaining informed consent. As an incentive, patients received a report summarizing their individual vulnerability and their efforts regarding heat-related health issues. This report was designed to support the patients’ consultations with their GPs about heat and was sent after the survey was completed.

### Ethics Considerations

The CLIMATE study was approved by the local Psychological Ethics Committee at the Center for Psychosocial Medicine of the University Medical Center Hamburg-Eppendorf (reference number LPEK-0605, approval date: May 1, 2023) and was prospectively registered on ClinicalTrials.gov (reference number NCT05961163). The study is reported in accordance with the Checklist for Reporting Results of Internet E-Surveys (CHERRIES) [15].

### Data Collection

#### Overview

The online survey was conducted using the established online survey tool Inquery (Inworks GmbH). After documenting informed consent within the tool, participants received an email containing a link to access the baseline assessment. They subsequently received invitations for up to 7 assessments of symptoms via email from July 24 to September 3, 2023. Each invitation was sent at 6 PM. Each specific link was associated with a unique data set to prevent duplicate entries. No cookies, IP checks, time stamps, or log file analyses were used. Incomplete questionnaires were deleted before analysis.

The baseline questionnaire included 85 items, while the assessment of symptoms comprised 18 items. Each page in the survey tool contained only 1 item. Answering all items was mandatory, but adaptive questioning (eg, filter questions) was used, and nonresponse options (eg, “not applicable”) were provided. The items in the survey were not randomized or altered between participants. Study participants could review and modify their answers before completing each questionnaire.

Specific days of observation were selected by the study team based on the maximum temperature expected during the respective weeks. The weather forecast was checked every Friday. If the forecast indicated that the maximum temperature would exceed 30°C in the upcoming 4 days, the warmest day within that period was chosen. Otherwise, the weather forecast was checked again on Tuesday to select the warmest of the remaining days in the week. In weeks with more than 1 hot day, multiple observations were possible. Observations were excluded if patients reported that they had not been at home during the respective time frame.

#### Outcome Variable: Severity of Symptoms

The endpoints of the study were health complaints measured using a symptom diary at each follow-up assessment. Monitored complaints were tiredness/fatigue, depressive symptoms, shortness of breath, dizziness, circulatory problems/loss of consciousness, edema (eg, in the legs), headache, nausea, anxiety, muscle cramps, confusion, palpitations, extrasystole, and vomiting.

The participants rated the highest severity in each symptom category over the last 24 hours based on the degree of limitations in their activities, using a 5-point Likert scale (ie, 0=“none,” 1=“light, not limiting,” 2=“moderate, limiting normal activities,” 3=“severe, stopping normal activities,” and 4=“very severe, stopping [almost] all activities”). A summary score considering all 14 symptom categories was calculated as a continuous outcome variable.

#### Independent Variable: Heat Exposure

For each day of observation, hourly local climate data were extracted from the website of Germany’s National Meteorological Service, Deutscher Wetterdienst [16], using the measuring station nearest to each participant’s home, as determined by postal code. Heat exposure was modeled by the maximum heat index on the day of observation, calculated from temperature in °C (*t*) and humidity in % (*h*) using the published formula [17-19]:

Heat index = –8.784695 + 1.61139411 × t + 2.338549 × h – 0.14611605 × t × h – 0.012308094 × t^2^ – 0.016424828 × h^2^ + 0.002211732 × t^2^ × h + 0.00072546× t × h^2^ + –0.000003582 × t^2^× h^2^

If participants did not complete the symptom diary on the day the follow-up invitation was sent, we used the following data:

- Data from the preceding day (if the assessment was made before 2 PM)
- Data from either the day of observation or the preceding day, depending on which day had a higher maximum temperature (if the assessment was made between 2 PM and 6 PM)
- Data from the day of observation (if the assessment was made at 6 PM or later).

The heat index was analyzed in 3 categories: colder weather (≤27°C), light heat (>27°C to 32°C), and moderate heat (>32°C to 36°C) [19].

#### Independent Variable: Efforts Against the Adverse Effects of Heat

Efforts to mitigate the adverse effects of heat were measured using a self-developed and validated questionnaire. After extracting 24 relevant items from the literature on heat, health literacy, social support, and resilience [8-11,20-22], an expert panel selected relevant items and refined them as necessary to optimize content validity. The expert panel comprised 8 clinicians from various medical specialties, including general medicine, geriatrics, emergency medicine, cardiology, diabetology, nephrology, neurology/psychiatry, and pulmonology.

In the first step, each item was rated by the experts in the categories of relevance, strength of evidence, comprehensibility, and potential to be influenced, using a 3-point scale (–1=low, 0=medium, and 1=high). A summary score was calculated for each item, and those with a mean score of 0.5 points or less were excluded from the questionnaire. Experts were also encouraged to provide comments and suggestions for modifying existing items or adding new ones. In the second step, the revised questionnaire was reassessed by the experts, resulting in further comments and suggestions. The final version of the questionnaire was then implemented in the baseline assessment of the online survey.

#### Other Independent Variables

The baseline questionnaire also included sociodemographic data, details about the participants’ chronic diseases, and standardized instruments for assessing health literacy, self-efficacy, and somatosensory amplification. Sociodemographic data comprised age, sex, living arrangement, educational level, and the country of birth of participants and their parents. Educational level was categorized according to the Comparative Analysis of Social Mobility in Industrial Nations Classification (CASMIN), based on the highest attained general and vocational qualifications in tertiary, secondary, and primary education or below [23]. The spectrum of observed chronic diseases reflected the inclusion criteria. Diseases were assessed using patient-oriented language.

General health literacy was assessed using the 16-item European Health Literacy Questionnaire (HLS-EU-Q16) [24,25]. After data collection, the 4-point Likert scale for each item in this questionnaire was dichotomized (agree=1, do not agree=0). We used the General Self-Efficacy Scale to determine the self-efficacy of the participants [26,27]. Additionally, the Somatosensory Amplification Scale was used to measure the tendency to catastrophize harmless bodily sensations, such as hunger, insect bites, or the sound of one’s own heartbeat [28,29]. For all scales, a summary score was calculated.

### Statistical Analysis

Descriptive data for categorical variables were reported as counts and percentages, while continuous variables were described using means and SDs. Additionally, the distribution of continuous independent variables was illustrated with histograms. Associations between independent variables were analyzed using a Pearson correlation matrix, and associations between the severity of reported symptoms and chronic disease clusters were determined using 2-sided (unpaired) *t* tests.

The structural validity of the questionnaire assessing efforts against the adverse effects of heat was tested using exploratory factor analysis with the principal factors method. Factors with an eigenvalue of 1.0 or higher were extracted, and orthogonal varimax rotation was applied. Items with loadings of less than 0.3 on any extracted factor were excluded. Based on the resulting factor structure, items were assigned to subscales.

We conducted mixed effects multivariable linear regression analyses, adjusted for random effects on the climate measuring station level and the study participant within the climate measuring station level, to analyze the association between independent variables and the summary score of self-reported symptoms (outcome). The analysis utilized the available data set and was controlled for the time of observation. We used the natural sample without weighting or propensity scores.

Associations between independent variables and the outcome were assessed using 3 nested statistical models. Model 1 included sociodemographic data and chronic diseases as independent variables. Model 2 expanded on model 1 by adding efforts against the adverse effects of heat and health literacy scores. Model 3 further included self-efficacy and somatosensory amplification scores. Improvements in model fit between the models were determined using the likelihood ratio test.

Additionally, we calculated unadjusted associations between independent variables and the summary score of self-reported symptoms, controlling for time of observation and for random effects at both the climate measuring station level and the participant level within each climate measuring station. An α level of 5% (*P*<.05) was defined as statistically significant. Statistical analyses were conducted using Stata 15.1 (StataCorp).

## Results

### Participants

**Figure 1:**
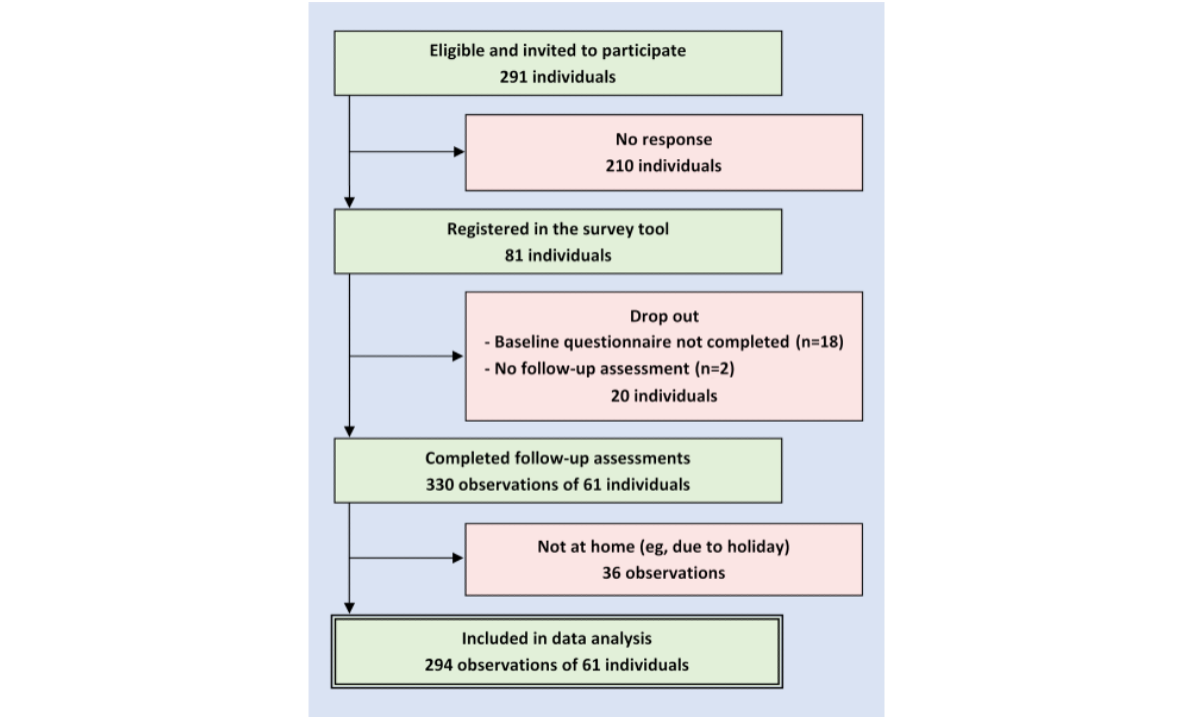
Recruitment of participants.

A total of 14 GP practices participated in the project, with 10 located in Northern Germany and 4 in Southern Germany. The recruitment of participants for the online survey is illustrated in Figure 1. Of the 291 eligible patients invited to participate, 81 registered in the survey tool, and 61 (21.0%) completed both the baseline assessment and 1 or more follow-up assessments. A total of 361 invitations for follow-up assessments were sent, and 330 (91.4%) observations were reported by participants. However, 36 observations had to be excluded because participants indicated that they were not in their hometown on the day of observation.

**Figure 2:**
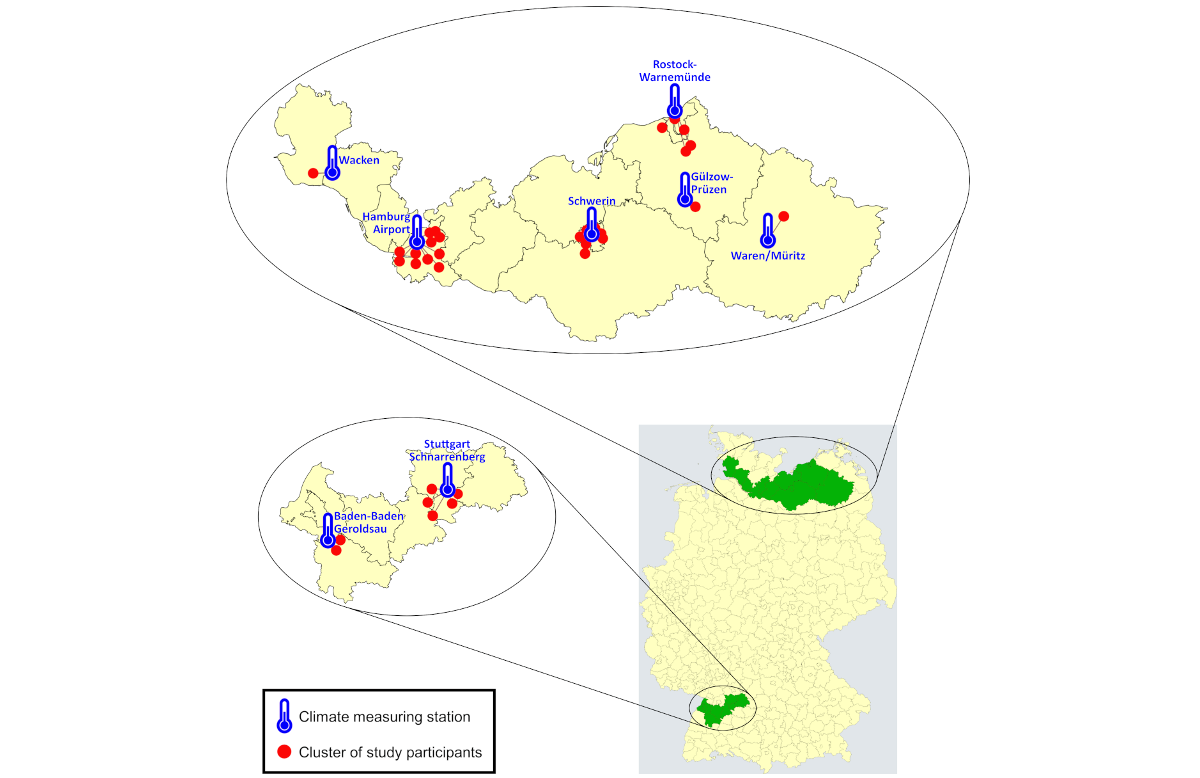
Location of study participants (n=61) clustered by postal code and corresponding climate measuring stations.

Consequently, the final sample size for data analysis was 294 observations from 61 participants.Figure 2 shows the locations of study participants clustered by postal code, along with the corresponding climate measuring stations.

### Efforts Against the Adverse Effects of Heat Questionnaire

The results of the expert panel are displayed in Figure S1A-E in Multimedia Appendix 1; 13 out of 24 items were excluded because the mean summary score of the expert ratings was 0.5 points or less. A total of 7 items were included without modification, while 4 items were included after being modified based on the experts’ comments. Additionally, 1 excluded item was revised and reincluded following suggestions from 2 experts in the second round of the panel.

The final questionnaire comprised 12 items. No floor or ceiling effects were observed in the distribution of the items, which exhibited a wide variance in difficulty. For instance, the majority of participants (58/61, 95%) either completely or largely agreed with the statement, “On warm days, I put on short, thin, or airy clothes.” By contrast, only 18 participants (30%) either completely or largely agreed with the statement, “When it gets extremely hot, others worry about me and ask how I am” (see Figure S2 in Multimedia Appendix 1).

The results from the factor analysis and rotation of efforts against the adverse effects of heat items are presented in Table S1 in Multimedia Appendix 1. Two factors with eigenvalues of 1 or more were extracted: health literacy (factor 1) and health behavior (factor 2). Three items had loadings ranging from 0.580 to 0.702 on the health literacy factor, while 5 items had loadings between 0.372 (inverted) and 0.552 on the health behavior factor. Four items had factor loadings of less than 0.3 on both extracted factors and were therefore excluded from the analysis.

### Baseline Data

Sociodemographic data and health status of participants at baseline are presented in Table 1. The mean age of participants was 61 (SD 14) years, with 31 (51%) identifying as men. A total of 15 participants (25%) lived alone, while 25 (41%) had attained tertiary education and 4 (7%) were born abroad. The most prevalent chronic diseases among participants (N=61) were cardiovascular diseases (n=23, 38%) and diabetes mellitus (n=20, 33%). Histograms of continuous baseline variables can be found in Figure S3 in Multimedia Appendix 1. The Subscale for Health Behavior in the Efforts Against the Adverse Effects of Heat Questionnaire had a mean score of 9.8 (SD 2.4) points, while the Health Literacy Subscale had a mean score of 5.0 (SD 2.3) points. The General Health Literacy Questionnaire yielded a mean score of 11.9 (SD 3.5) points, the General Self-Efficacy Scale had a mean of 29.9 (SD 5.3) points, and the Somatosensory Amplification Scale reported a mean score of 17.4 (SD 6.2) points.

A correlation matrix of independent variables is presented in Table S2 in Multimedia Appendix 1. Excluding associations between dummies of the same categorical variable, we identified several correlations among independent variables: older participants had a higher probability of cardiovascular disease (*r*=0.46) and reported better health behavior (*r*=0.35). Participants living alone reported worse general health literacy (*r*=–0.31), while those with psychiatric disorders exhibited lower general self-efficacy (*r*=–0.46). Additionally, there was a positive association between general self-efficacy and general health literacy (*r*=0.42), as well as a positive relationship between somatosensory amplification and self-reported health behavior according to the Efforts Against the Adverse Effects of Heat Scale (*r*=0.38).

**Table 1:** Sociodemographic data and health status (N=61).

Characteristic				Value
Age (years), mean (SD)				61 (14)
**Sex, n (%)**				
	Men			31 (51)
	Women			30 (49)
**Living arrangement, n (%)**				
	Living alone			15 (25)
	**Living together with others**			46 (75)
		Married or cohabiting	40 (66)	
		Together with own children or children of partner	12 (20)	
		Together with own parents or parents of partner	3 (5)	
		Together with other family members	2 (3)	
		Together with others (eg, in a shared flat)	4 (7)	
**Educational level, n (%)**				
	Tertiary			25 (41)
	Secondary			27 (44)
	Primary or below			9 (15)
**Country of birth, n (%)**				
	Participants and parents born in Germany			48 (79)
	Participants born in Germany, at least one parent abroad			9 (15)
	Participants born abroad			4 (7)
**Chronic diseases, n (%)**				
	**Cardiovascular diseases**			23 (38)
		Myocardial infarction	9 (15)	
		Coronary heart disease	9 (15)	
		Cardiac arrhythmias (medication or pacemaker)	8 (13)	
		Heart failure (at least slight limitations during ordinary activity)	7 (11)	
		Stroke or transitory ischemic attack	4 (7)	
	Diabetes mellitus type 1 or 2 (oral medication or insulin)			20 (33)
	**Respiratory diseases** **, n (%)**			11 (18)
		Asthma (daily treatment)	8 (13)	
		Chronic obstructive pulmonary disease (oral medication)	3 (5)	
	**Psychiatric disorders** **, n (%)**			10 (16)
		Depression (treated with medication)	8 (13)	
		Anxiety (treated with medication)	2 (3)	
		Schizophrenia (treated with medication)	1 (2)	
	Neuropathy (regularly or persistently occurring symptoms), n (%)			4 (7)
	Renal insufficiency (medication or dialysis), n (%)			2 (3)

### Follow-Up Data

The mean symptom burden severity score was 5.4 (SD 5.0) points, as illustrated in the histogram shown in Figure S4 in Multimedia Appendix 1. Significant differences in reported severity were observed among chronic disease clusters. Participants with renal insufficiency reported substantially higher symptom severity scores (10.7 vs 5.16, *P*<.001) compared with those without. Similarly, participants with psychiatric disorders had higher scores (8.06 vs 4.92, *P*<.001). By contrast, participants with cardiovascular diseases reported lower severity (3.52 vs 6.71, *P*<.001), as did those with diabetes mellitus (4.03 vs 6.14, *P*=.001). These differences were also evident in the specific symptoms, as detailed in Table S3 in Multimedia Appendix 1.

**Figure 3:**
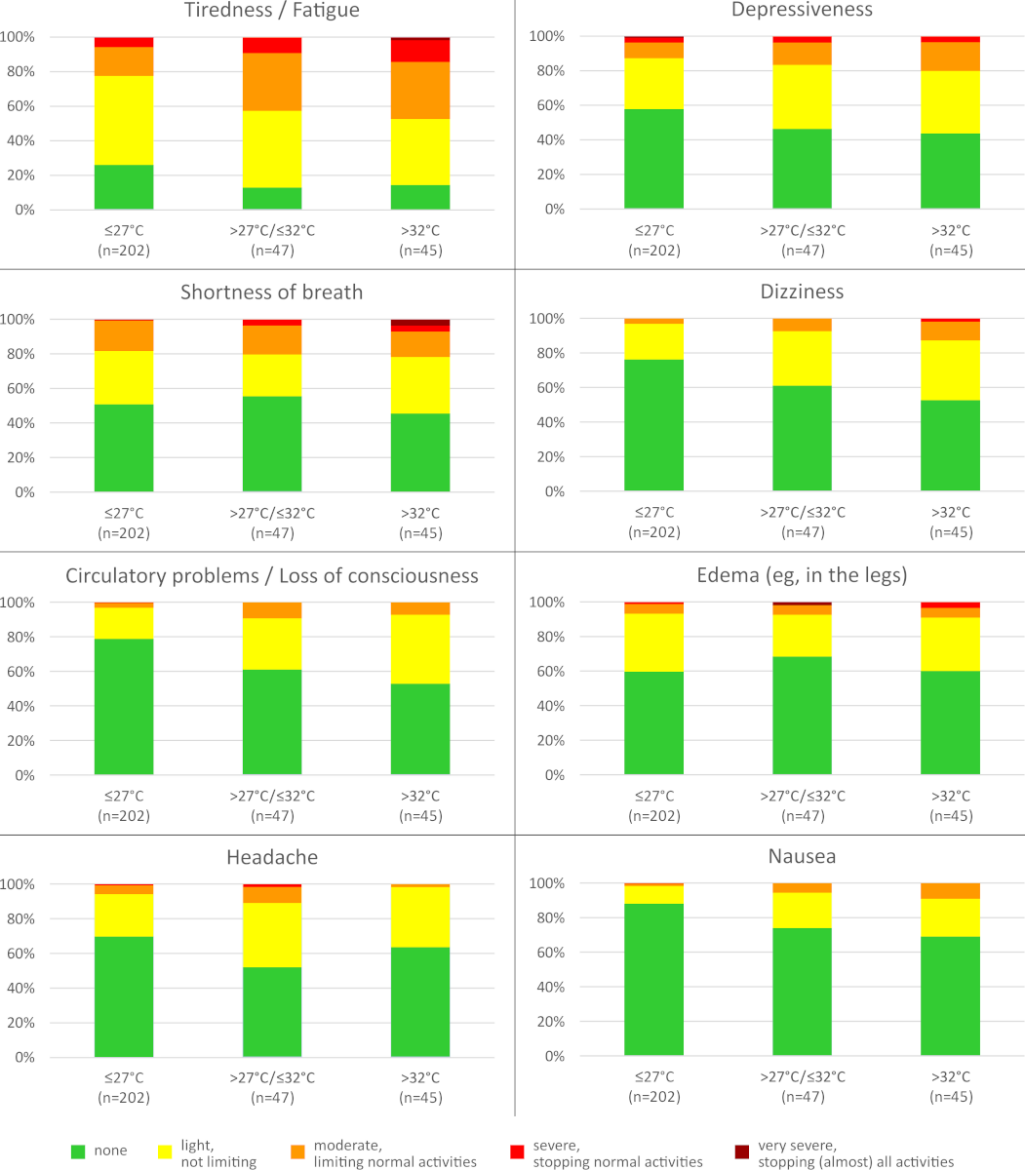
Reported symptoms (n=294).

On average, the climate measuring stations recorded a heat index of 26.8°C (SD 3.3°C) on the observation days. The mean temperatures across the measuring stations are presented in Figure S5 in Multimedia Appendix 1. The symptom severity score increased with the heat index: it was 4.8 (SD 4.6) at temperatures of 27°C or less, 6.4 (SD 5.9) for temperatures between 27°C and 32°C, and 7.0 (SD 5.2) at temperatures exceeding 32°C. The prevalence of specific symptoms corresponding to the heat index is illustrated in Figure 3 and Figure S6 in Multimedia Appendix 1. For the symptoms of tiredness/fatigue, depressiveness, shortness of breath, dizziness, circulatory problems/loss of consciousness, nausea, confusion, and vomiting, there is a slight to moderate increase in severity with higher heat index categories. By contrast, the other 5 symptom categories appear to be relatively independent of the observed risk levels.

### Association Between Independent Variables and Outcome

The results of the multivariable models examining the association between independent variables and symptom burden are presented in Table 2. The results of the unadjusted models are available in Tables S4-S13 in Multimedia Appendix 1.

**Table 2:** Association of symptom burden with heat, sociodemographic data, health status, efforts against the adverse effects of heat, health literacy, self-efficacy, and somatosensory amplification: results of multivariable linear regression adjusted for random effects on the levels of climate measuring stations and patients (n=294).

Characteristics		Model 1		Model 2		Model 3	
		β (95% CI)	*P* value^a^	β (95% CI)	*P* value^a^	β (95% CI)	*P* value^a^
**Heat index risk level**							
	≤27°C	*Reference*	*Reference*	*Reference*	*Reference*	*Reference*	*Reference*
	>27°C-32°C	*1.02 (0.08 to 1.96)*	*.03*	*1.07 (0.13 to 2.01)*	*.03*	*0.98 (0.05 to 1.91)*	*.04*
	>32°C	*1.35 (0.35 to 2.35)*	*.008*	*1.44 (0.44 to 2.43)*	*.005*	*1.35 (0.37 to 2.33)*	*.007*
Age		0.04 (–0.03 to 0.12)	.25	0.01 (–0.06 to 0.08)	.84	0.02 (–0.04 to 0.07)	.58
**Sex**							
	Men	Reference	Reference	Reference	Reference	Reference	Reference
	Women	1.18 (–0.73 to 3.09)	0.23	0.31 (–1.44 to 2.06)	.73	–0.42 (–1.87 to 1.02)	.57
**Living arrangement**							
	Living together with others	Reference	Reference	Reference	Reference	Reference	Reference
	Living alone	–0.29 (–2.35 to 1.78)	0.78	0.14 (–1.79 to 2.08)	.88	–0.33 (–1.92 to 1.27)	.69
**Educational level**							
	Tertiary	*Reference*	*Reference*	Reference	Reference	*Reference*	*Reference*
	Secondary	1.54 (–0.42 to 3.50)	.12	1.14 (–0.57 to 2.85)	.19	*2.23 (0.80* *to* *3.66)*	*.002*
	Primary or below	*3.54 (0.90* *to* *6.17)*	*.008*	2.05 (–0.35 to 4.45)	.09	*2.59 (0.63* *to* *4.55)*	*.009*
**Country of birth**							
	Participant and parents born in Germany	Reference	Reference	Reference	Reference	Reference	Reference
	Participant born in Germany, at least one parent abroad	0.80 (–1.65 to 3.25)	.52	1.41 (–0.73 to 3.54)	.20	0.46 (–1.29 to 2.22)	.60
	Participant born abroad	–0.61 (–4.21 to 3.00)	.74	–0.50 (–3.69 to 2.69)	.76	–1.47 (–4.07 to 1.13)	.27
**Chronic diseases**							
	Cardiovascular diseases	–*2.54 (–4.67* *to* *–0.40)*	*.02*	–*2.57 (–4.42 to –0.72)*	*.006*	–*2.46 (–3.99* *to* *–0.93)*	*.002*
	Diabetes mellitus	–*2.99 (–4.92* *to* *–1.06)*	*.002*	–*3.38 (–5.05 to –1.71)*	*<.001*	–*3.02 (–4.37* *to* *–1.66)*	*<.001*
	Respiratory diseases	–0.37 (–2.81 to 2.06)	.76	–0.66 (–2.79 to 1.47)	.55	–0.88 (–2.61 to 0.85)	.32
	Psychiatric disorders	1.63 (–0.94 to 4.20)	.21	1.30 (–0.95 to 3.56)	.26	–1.23 (–3.27 to 0.81)	.24
	Neuropathy	0.36 (–3.28 to 4.00)	.85	–0.60 (–3.78 to 2.57)	.71	–0.33 (–2.91 to 2.26)	.81
	Renal insufficiency	4.04 (–0.71 to 8.79)	.10	*4.30 (0.17 to 8.43)*	*.04*	1.74 (–1.70 to 5.18)	.32
**Efforts against the adverse effects of heat**							
	Subscale Health Behavior	N/A^b^	N/A	*0.65 (0.29 to 1.00)*	*<.001*	0.31 (–0.005 to 0.62)	.05
	Subscale Health Literacy	N/A	N/A	–0.03 (–0.36 to 0.29)	.83	–0.09 (–0.36 to 0.18)	.50
Health literacy		N/A	N/A	–*0.25 (–0.49 to –0.01)*	*.04*	0.002 (–0.21 to 0.22)	.99
General self-efficacy		N/A	N/A	N/A	N/A	–*0.39 (–0.54* *to* *–0.23)*	*<.001*
Somatosensory amplification		N/A	N/A	N/A	N/A	*0.18 (0.07* *to* *0.28)*	*.001*

^a^Statistically significant results (*P*≤.05) are shown in italics.

^b^N/A: not applicable.

### Multivariable Models

In model 1, compared with a heat index of 27°C or less, a heat index greater than 27°C but less than or equal to 32°C (β=1.02, 95% CI 0.08-1.96, *P*=.03) and a heat index greater than 32°C (β=1.35, 0.35-2.35, *P*=.008) were associated with a higher symptom burden. Primary education or below, compared with tertiary education (β=3.54, 0.90-6.17, *P*=.008), was also associated with a higher symptom burden, while cardiovascular diseases (β=–2.54, –4.67 to –0.40, *P*=.02) and diabetes mellitus (β=–2.99, –4.92 to –1.06, *P*=.002) were associated with a lower symptom burden.

In model 2, the effects of heat index (β=1.07, 95% CI 0.13-2.01, *P*=.03 for >27°C-32°C and β=1.44, 95% CI 0.44-2.43, *P*=.005 for >32°C), cardiovascular diseases (β=–2.57, 95% CI –4.42 to –0.72, *P*=.006), and diabetes mellitus (β=–3.38, 95% CI –5.05 to –1.71, *P*<.001) remained stable; however, tertiary education lost its statistical significance (*P*=.09). Instead, a higher score in the Health Behavior Subscale of the Efforts Against the Adverse Effects of Heat Questionnaire (β=0.65, 95% CI 0.29-1.00, *P*<.001) and a lower score in the General Health Literacy Scale (β=–0.25, 95% CI –0.49 to –0.01, *P*=.04) were associated with a higher symptom burden. Additionally, renal insufficiency (β=4.30, 95% CI 0.17-8.43, *P*=.04) was associated with a higher symptom burden. In model 2, there was a statistically significant improvement in model fit compared with model 1 (*P*=.002).

In model 3, the effects of heat index remained stable for a heat index between more than 27°C and 32°C (β=0.98, 95% CI 0.05-1.91, *P*=.04), and for a heat index of more than 32°C (β=1.35, 95% CI 0.37-2.33, *P*=.007). The impact of cardiovascular diseases was β=–2.46 (95% CI –3.99 to –0.93, *P*=.002) and diabetes mellitus was β=–3.02 (95% CI –4.37 to –1.66, *P*<.001). Additionally, the educational level regained statistical significance, with β=2.23 (95% CI 0.80-3.66, *P*=.002) for secondary education and β=2.59 (95% CI 0.63-4.55, *P*=.009) for primary education or below compared with tertiary education. However, the General Health Literacy Scale (*P*=.99) and the Health Behavior Subscale of the Efforts Against the Adverse Effects of Heat Questionnaire (*P*=.05) lost their statistical significance. Instead, a lower score on the General Self-Efficacy Scale (β=–0.39, 95% CI –0.54 to –0.23, *P*<.001) and a higher score on the Somatosensory Amplification Scale (β=0.18, 0.07-0.28, *P*=.001) were associated with a higher symptom burden. Additionally, in model 3, there was a statistically significant improvement in model fit compared with model 2 (*P*<.001).

### Unadjusted Analyses

With the exception of cardiovascular diseases, all associations identified in the multivariable models were replicated. These included primary education or below compared with tertiary education (β=4.19, 95% CI 1.23-7.16, *P*=.006), diabetes mellitus (β=–2.61, 95% CI –4.61 to –0.60, *P*=.01), renal insufficiency (β=5.85, 95% CI 0.90-10.80, *P*=.02), the Health Behavior Subscale of the Efforts Against the Adverse Effects of Heat Questionnaire (β=0.64, 95% CI 0.24-1.04, *P*=.002), general health literacy (β=–0.34, 95% CI –0.63 to –0.05, *P*=.02), general self-efficacy (β=–0.52, 95% CI –0.67 to –0.37, *P*<.001), and somatosensory amplification (β=0.25, 95% CI 0.09-0.41, *P*=.002).

Additionally, statistically significant associations were found between the female sex (β=2.16, 95% CI 0.14-4.18, *P*=.04) and neuropathy (β=3.01, 95% CI 0.45-5.57, *P*=.02) with a higher symptom burden. In all unadjusted analyses, a heat index of more than 32°C (β=1.33, 95% CI 0.33-2.32, *P*=.009 through β=1.43, 95% CI 0.42-2.43, *P*=.005) and, in 9 of 10 unadjusted analyses, a heat index between more than 27°C and 32°C (β=0.98, 95% CI 0.03-1.92, *P*=.04 through β=1.02 and 95% CI 0.08-1.96, *P*=.03) compared with a heat index of 27°C or less were associated with a higher symptom burden.

## Discussion

### Principal Findings

Compared with colder weather, light and moderate heat were associated with more severe health complaints among chronically ill patients in their everyday lives. Adverse effects were less severe when participants had higher self-efficacy and lower somatosensory amplification, the latter meaning they were less likely to catastrophize normal bodily sensations. Contrary to our hypothesis, self-reported efforts to mitigate the adverse effects of heat were not associated with less severe health complaints in our study. Instead, we found that somatosensory amplification mediated the effect of self-reported protective behavior on symptom severity. Specifically, patients who were more likely to catastrophize their bodily sensations reported both greater efforts to mitigate the adverse effects of heat and higher symptom severity simultaneously.

### Strengths and Limitations

In our pilot study, the feasibility of the study design was demonstrated. Despite a low sample size of 61 participants and 294 observations, the statistical power of the study was sufficient to detect many strong associations within the data set. Participants in our online survey were recruited by the staff of cooperating general practices based on defined inclusion and exclusion criteria, which likely represent the target population better than self-selection [30,31]. Another strength of the study was the high data quality, including a valid operationalization of heat, no missing values in the survey data, and a high rate (330/361, 91.4%) of completion of follow-up assessments. All necessary weather data were available and could be merged with the survey data. Participants from both urban and rural regions in Northern and Southern Germany were included, providing a variation of more than 7°C in mean temperature.

Further strengths of the study are the selected assessment instruments. Heat and humidity were represented by data from measuring stations of the German National Meteorological Service, which are the best available data sources. The Efforts Against the Adverse Effects of Heat Questionnaire was developed based on the literature, and we enhanced the content validity of the instrument through an expert panel that selected relevant items and refined the wording. Moreover, the structural validity of this questionnaire has been demonstrated through exploratory factor analysis. The other analyzed constructs were measured using validated and well-established questionnaires [24-29]. Our data were analyzed using robust statistical methods that adjusted for confounding and cluster effects, and the results from the multivariable models were confirmed in the unadjusted analyses. Furthermore, health complaints were assessed on the respective day, making recall bias regarding the outcome variable unlikely.

One limitation of the study is the low participation rate (61/291, 21.0%). This may have affected the representativeness of the sample; however, associations detected in a data set are usually independent of the participation rate [32-35]. It is also possible that additional independent variables are associated with the outcome variable but could not be detected due to the low statistical power of our pilot study. For example, we found no association between living arrangements and adverse effects of heat. This could indicate that these variables are independent of each other or that the statistical power was insufficient to detect a difference. Additionally, we excluded individuals with insufficient German language skills, so we do not know whether they are similarly affected as German-speaking individuals. As a result of the unusually low temperatures during the observation period, participants were exposed only to light to moderate heat. Thus, the effects of heat, protective behavior, and the other measured constructs may have been underestimated.

Unfortunately, it is not possible to calculate exact measures of the sample’s representativeness because we have very specific inclusion criteria (eg, the selected chronic diseases), and there are significant differences in sociodemographic data across regions. However, when compared with the overall general population in Germany, our study appears to be relatively representative regarding the percentage of women (30/61, 49%, in our study vs 51% in the general population, as published by Statistisches Bundesamt [36]), individuals with a migration background (13/61, 21%, vs 26%), and 1-person households (15/61, 25%, vs 20%). There are, however, notable differences in educational levels between the sample and the general population. In Germany, less than 20% of individuals have received tertiary education, while 50% have attained secondary education and 30% have primary education or below. In our sample, the rate of tertiary education is significantly higher (25/61, 41%), whereas the rate of primary education or below is much lower (9/61, 15%) [36]. The uneven representation of educational levels may stem from the decision to conduct an online survey instead of interviews or postal surveys. Consequently, the presented results may not be representative of individuals who lack access to or prefer not to use online surveys.

Another consequence of the low sample size was that, compared with the prospective study registration, the endpoints and statistical methods had to be slightly simplified. Therefore, we analyzed a summary score of symptom severity using multivariable multilevel linear regression instead of a composite index reflecting the highest severity in any category, which would have been analyzed using multivariable multilevel ordinal logistic regression. Moreover, due to comparably low average temperatures, only 7 weeks of observation instead of the planned 12 were completed. Additionally, multiple invitations to follow-up were sent each week if more than 1 hot day was anticipated. Aside from these adjustments, the study design was conducted as per the prospective registration.

### Comparison With the Literature

Recently, an increasing number of publications have addressed the possible adverse effects of heat on the human body [37,38]. Most of these studies have focused on mortality or hospital admissions [3,39,40]. Some meta-analyses have considered small changes in ambient temperature [41,42]. Increased indoor temperatures are associated with higher symptom levels in older individuals and in those with cardiovascular or respiratory diseases [43-45]. One study found that adaptive measures are taken during heat events [46], but it was not elaborated whether these measures reduce adverse health effects.

Our findings suggest that individuals with cardiovascular diseases or diabetes mellitus experience fewer adverse effects during light and moderate heat compared with those with other heat-related chronic diseases. This observation appears contrary to recent literature, which identifies individuals with cardiovascular diseases as having the highest mortality rates due to a greater likelihood of cardiovascular events, such as myocardial infarction or cardiac arrest [4,47,48], which our study did not address. It is worth noting that the effect of heat on congestive heart failure is debatable, as one meta-analysis suggested that heat may be beneficial for this specific condition [49]. The impact of heat on individuals with diabetes may depend on blood sugar management [50,51], which we did not monitor in our population.

The adverse effects we found in individuals with chronic kidney disease align with those reported in other studies. The impact of heat is mediated by dehydration, which leads to further kidney damage and contributes to the development of cardiovascular events [52-54]. Our findings regarding respiratory and mental health issues also appear to reflect the existing literature. Across multiple studies, the symptom burden was exacerbated by poorer air quality, increasing amounts of fine particulate matter, and elevated cell death, which causes a heightened immune response [5,55,56]. The mechanism of action regarding the effect of heat on individuals with mental illness remains unclear. Nonetheless, individuals suffering from mental illness are significantly affected by higher ambient temperatures, leading to increased mortality and symptom burden [57-59].

In our study, self-reported protective behavior at baseline was not associated with a decreased symptom burden during follow-up. Studies on protective behavior during heat waves have produced mixed results regarding adherence to protective measures following public information campaigns. In some studies, vulnerable subgroups adhered to protective measures [60,61], while in others, vulnerable populations could not be adequately reached [62-65]. One study found that owning and using air conditioning can reduce the impact of heat on health complaints [66]. However, there is still little evidence regarding whether other protective behaviors reduce the symptom burden associated with high temperatures.

### Implications for Clinical Practice and Research

It remains unclear whether retrospective self-reports are an appropriate tool for assessing health behavior during heat. Evidence suggests that self-reported behavior often does not align with actual behavior across different disciplines [67-69]. For instance, there is a significant gap between self-reported and measured physical activity, ranging up to 50% [70,71]. It is possible that the intention-behavior gap observed in sports science [72] also applies to protective behavior against the adverse effects of heat [73]. Therefore, future studies should assess self-reported behavior on days of high temperatures, if possible, paired with the assessment of biodata such as blood pressure, heart rate, body temperature, and activity levels.

In our study, patients with high levels of somatosensory amplification reported better protective behavior and, at the same time, a higher symptom burden, which aligns with the literature. Other studies found an elevated symptom burden for patients with higher scores on the Somatosensory Amplification Scale related to pain sensation [74], as well as other symptoms such as respiratory symptoms [75] and gastrointestinal conditions [76]. Furthermore, patients with high scores in somatosensory amplification appear to seek more GP consultations [77] and report a healthier lifestyle [78]. Therefore, studies investigating the association between self-reported and actual protective behavior should also focus on potential interactions with somatosensory amplification.

Self-efficacy appears to be a protective factor against the adverse effects of heat, which is consistent with current literature. For instance, higher self-efficacy is linked to improved quality of life in breast cancer survivors [79], patients with hypertensive nephropathy [80], and active aging [81]. Health literacy also seems to be a protective factor; for example, health literacy correlates with the incidence of cardiovascular diseases [82], glucose levels in individuals with type 2 diabetes [83], medication adherence [84], and overall health status [85].

Medical staff can play a vital role in strengthening health literacy and self-efficacy, for example, using longer periods of individual consultation combined with group interventions [86,87]. However, it remains unknown whether such interventions can protect patients with chronic diseases from the adverse effects of heat. One study found telemonitoring to be effective in reducing exacerbations of chronic obstructive pulmonary disease during hot temperatures [88]. Nevertheless, further research is needed to evaluate which specific behavioral changes effectively reduce symptom burden during high temperatures and how these changes in health behavior can be facilitated.

### Conclusions

Compared with colder weather, light and moderate heat were associated with more severe health complaints. Symptom burden was lower among participants with higher self-efficacy and less somatosensory amplification. Self-reported protective behavior was not linked to lower symptom burden. Instead, we found that patients who were more prone to catastrophizing normal bodily sensations reported better protective behavior while also experiencing a higher symptom burden.

It remains unclear whether retrospective self-reports are an appropriate tool for assessing health behavior during heat. Future studies should evaluate self-reported behavior on days of high temperatures and focus on possible interactions with somatosensory amplification and self-efficacy. Further research is needed to determine which specific behavioral changes effectively reduce symptom burden during high temperatures and how these changes in health behavior can be facilitated.

## Appendix

Multimedia Appendix 1 Additional analysis results.

## Abbreviations

CASMIN: Comparative Analysis of Social Mobility in Industrial Nations Classification
CHERRIES: Checklist for Reporting Results of Internet E-Surveys
CLIMATE: Chronical Illness-Related Limitations of the Ability to Cope With Rising Temperatures
GP: general practitioner
HLS-EU-Q16: 16-item European Health Literacy Questionnaire
