# Supplementary material for: Association Between Self-Reported Protective Behavior and Heat-Associated Health Complaints Among Patients With Chronic Diseases in Primary Care: Results of the CLIMATE Pilot Cohort Study
Source: JMIR Public Health Surveill. 2024 Nov 4;10:e58711. doi: 10.2196/58711 (PMC11574497; doi:10.2196/58711)
Supplement: Multimedia Appendix 1 [file publichealth_v10i1e58711_app1.pdf]

ASSOCIATION BETWEEN SELF-REPORTED PROTECTIVE BEHAVIOUR AND HEAT-ASSOCIATED HEALTH COMPLAINTS AMONG PATIENTS WITH CHRONIC DISEASES IN PRIMARY CARE: RESULTS OF THE CLIMATE PILOT COHORT STUDY

Supplement 1: Additional results

Arne Jordan, Julia Nothacker, Valentina Paucke, Klaus Hager, Susann Hueber,
Arian Karimzadeh, Thomas Kötter, Christin Löffler, Beate S. Müller, Daniel Tajdar,
Dagmar Lühmann, Martin Scherer, and Ingmar Schäfer

Figure S1a: Results of expert panel of efforts against adverse effects of heat questionnaire

<table>
<tr><th>Original Item</th><th>Rating</th><th>Result of expert panel</th></tr>
<tr><td>Item 1: “When it gets extremely hot, others worry about me and ask me how I am.“</td><td>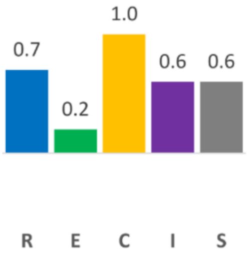
</td><td>Item included without modification:

“When it gets extremely hot, others worry about me and ask me how I am.“</td></tr>
<tr><td>Item 2: “In case of health problems, I can quickly get medical help in my place of residence.“</td><td>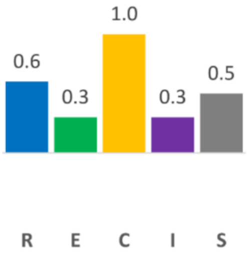
</td><td>Item excluded, because summary score was not > 0.5.</td></tr>
<tr><td>Item 3: “I don't have anyone who can support me (e.g. in the household). Even if I feel bad, I have to take care of my daily needs myself.“</td><td>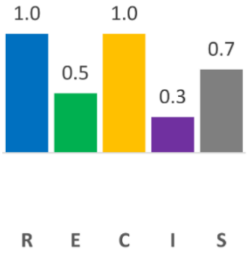
</td><td>Item included without modification:

“I don't have anyone who can support me (e.g. in the household). Even if I feel bad, I have to take care of my daily needs myself.“</td></tr>
<tr><td>Item 4: “I received advice from my family doctor on what to do in case of heat.“</td><td>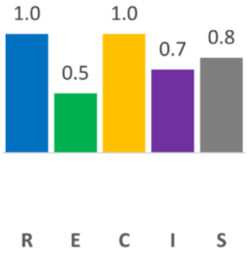
</td><td>Item included without modification:

“I received advice from my family doctor on what to do in case of heat.“</td></tr>
<tr><td>Item 5: “I find it hard to ask others for help when I need something.“</td><td>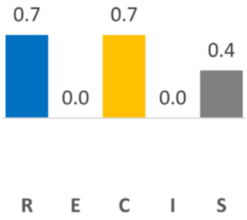
</td><td>Item excluded, because summary score was not > 0.5.</td></tr>
</table>

R: relevance; E: strength of evidence; C: comprehensibility; I: possibility to influence the item; S: Summary score.

Figure S1b: Results of expert panel of efforts against adverse effects of heat questionnaire (continued)

Original Item	Rating	Result of expert panel
Item 6: "There are health professionals I trust and who care about me."	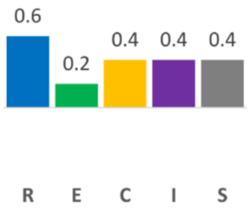	*Item excluded, because summary score was not > 0.5.*
Item 7: "Even on hot days there is a place in my apartment where the temperatures usually stay pleasant."	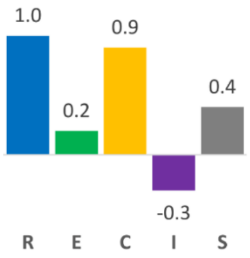	*Item excluded, because summary score was not > 0.5.*
Item 8: "When I am physically active outdoors on summer days, I am often exposed to direct sunlight (e.g. working in the garden, exercising, working in my job)."	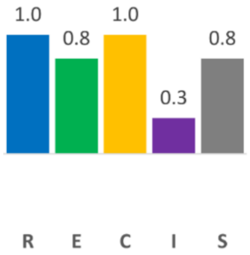	***Item included without modification:***<br><br>"When I am physically active outdoors on summer days, I am often exposed to direct sunlight (e.g. working in the garden, exercising, working in my job)."
Item 9: "Where I live, the air quality is often poor (e.g. low air circulation, many exhaust gases)."	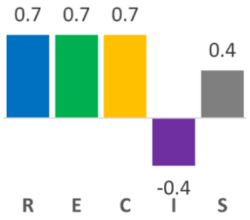	*Item excluded, because summary score was not > 0.5.*
Item 10: "When I'm on the road on sunny days at noon (e.g. visiting my doctor or going shopping), I usually stay in the shade."	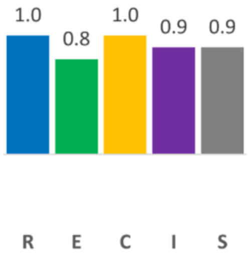	***Item included, but modified due to expert comments:***<br><br>"When I'm on the road on sunny days at noon (e.g. visiting my doctor or going shopping), I usually stay in the shade or use air-conditioned means of transport."

R: relevance; E: strength of evidence; C: comprehensibility; I: possibility to influence the item; S: Summary score.

Figure S1c: Results of expert panel of efforts against adverse effects of heat questionnaire (continued)

Original Item	Rating	Result of expert panel
Item 11: “I don't leave my apartment even if it’s very hot inside.“	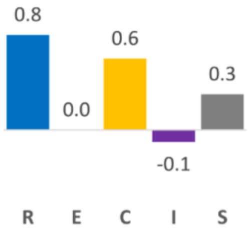	*Item excluded, because summary score was not > 0.5.*
Item 12: “On warm days, I prefer to visit cool places (e.g. garden, forest, shores of lakes or rivers, cool interiors).“	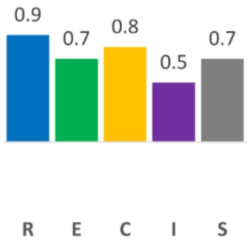	***Item included without modification:***<br><br>“On warm days, I prefer to visit cool places (e.g. garden, forest, shores of lakes or rivers, cool interiors).“
Item 13: “I'm rarely thirsty because I usually drink enough liquid.“	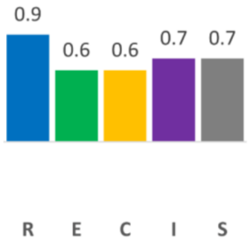	***Item included, but modified due to expert comments:***<br><br>“I always manage to drink enough liquid.“
Item 14: “When I have to do a job, I don't let headaches or mild nausea stop me.“	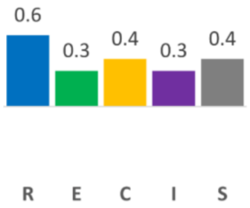	*Item excluded, because summary score was not > 0.5.*
Item 15: “On very hot days, I adjust my medication.“	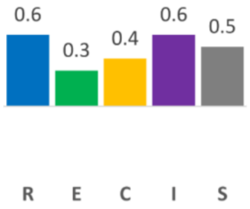	***Item excluded, because summary score was not > 0.5, but re-included and modified in stage 2 due to expert comments:***<br><br>“I know which medications I need to adjust in case of heat.”

R: relevance; E: strength of evidence; C: comprehensibility; I: possibility to influence the item; S: Summary score.

Figure S1d: Results of expert panel of efforts against adverse effects of heat questionnaire (continued)

Original Item	Rating	Result of expert panel
Item 16: "If it gets too warm, I make myself more comfortable with a fan, cold wraps or a foot bath."	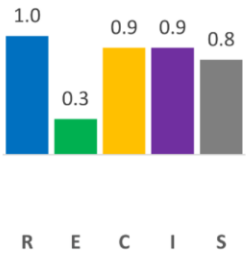	***Item included, but modified due to expert comments:***<br>"If it gets too warm, I make myself more comfortable with a fan/air conditioning, cold wraps or a foot bath."
Item 17: "In big heat I save myself and avoid physical activity."	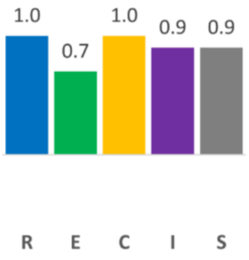	***Item included without modification:***<br>"In big heat I save myself and avoid physical activity."
Item 18: "I know what complaints I need to pay attention to when it's particularly hot."	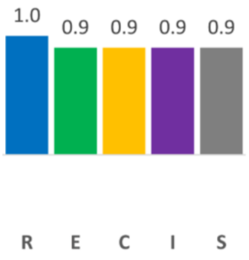	***Item included without modification:***<br>"I know what complaints I need to pay attention to when it's particularly hot."
Item 19: "On warm days I put on airy clothes."	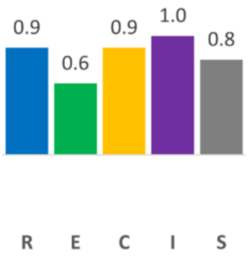	***Item included, but modified due to expert comments:***<br>"On warm days I put on short, thin or airy clothes."
Item 20: "I can usually manage the challenges of everyday life well."	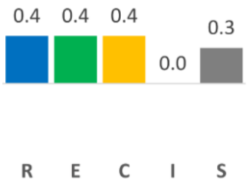	*Item excluded, because summary score was not > 0.5.*

R: relevance; E: strength of evidence; C: comprehensibility; I: possibility to influence the item; S: Summary score.

Figure S1e: Results of expert panel of efforts against adverse effects of heat questionnaire (continued)

Original Item	Rating	Result of expert panel
Item 21: "I don't get discouraged if I don't reach my goals."	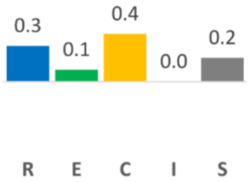	*Item excluded, because summary score was not > 0.5.*
Item 22: "Hot temperatures don't bother me."	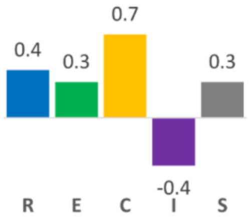	*Item excluded, because summary score was not > 0.5.*
Item 23: "I often plan something and then don't manage to do it."	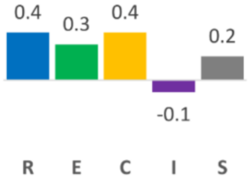	*Item excluded, because summary score was not > 0.5.*
Item 24: "I care about others and others also care about me."	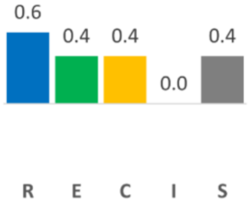	*Item excluded, because summary score was not > 0.5.*

R: relevance; E: strength of evidence; C: comprehensibility; I: possibility to influence the item; S: Summary score.

Figure S2: Efforts against adverse effects of heat questionnaire (n=61)

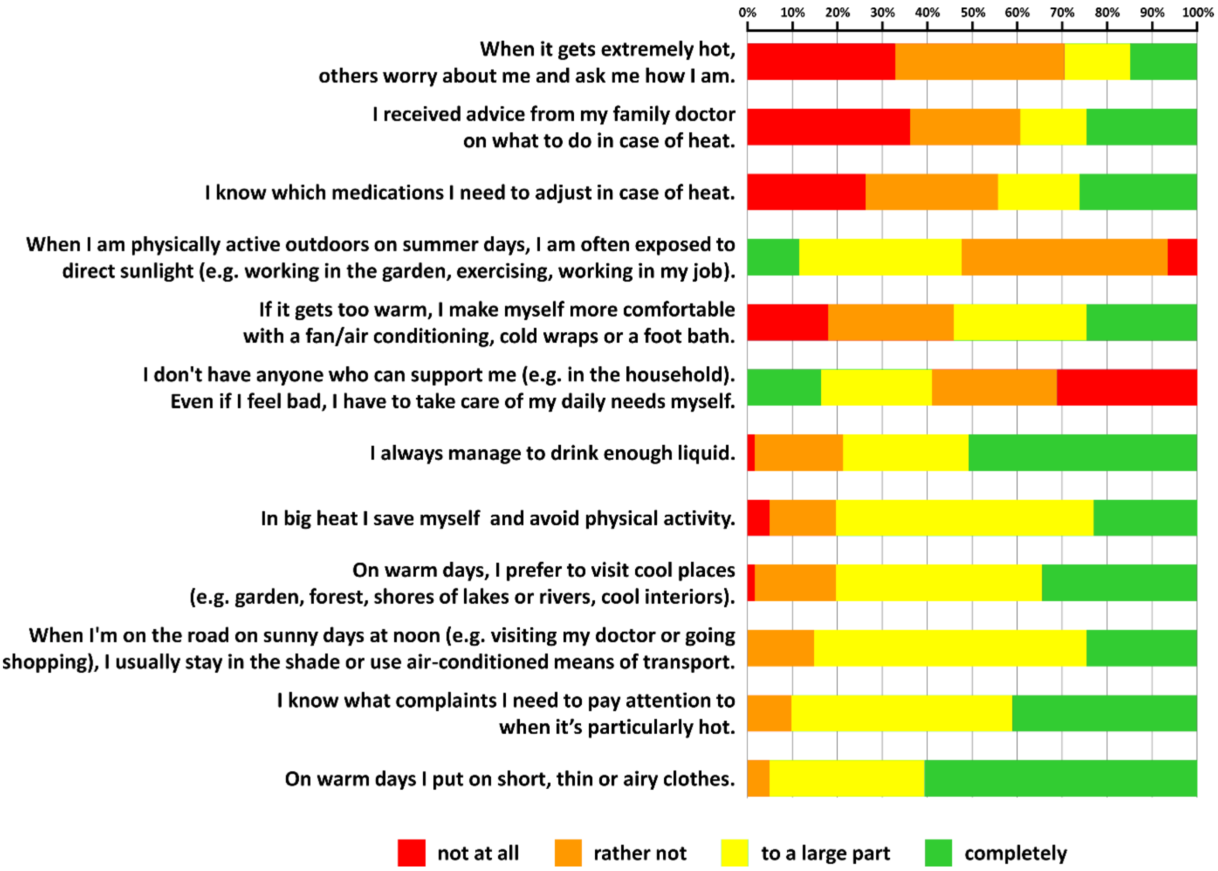

Reversed items have a reversed order of colours; n: number of participants.

Table S1: Efforts against adverse effects of heat questionnaire: Loadings of factors with eigenvalue ≥ 1, varimax-rotated (n=61)

	Factor 1: Health literacy	Factor 2: Health behavior
**Eigenvalue**	**1.371**	**1.216**
**Cumulative percent**	**0.476**	**0.909**
1. On warm days, I prefer to visit cool places (eg garden, forest, shores of lakes or rivers, cool interiors).	*	0.474
2. When it gets extremely hot, others worry about me and ask me how I am.	*	0.552
3. In big heat I save myself and avoid physical activity.	*	0.464
4. I always manage to drink enough liquid.	*	*
5. I received advice from my family doctor on what to do in case of heat.	0.580	*
6. When I am physically active outdoors on summer days, I am often exposed to direct sunlight (eg working in the garden, exercising, working in my job).	*	-0.371
7. On warm days I put on short, thin or airy clothes.	*	*
8. I don't have anyone who can support me (eg in the household). Even if I feel bad, I have to take care of my daily needs myself.	*	*
9. If it gets too warm, I make myself more comfortable with a fan/air conditioning, cold wraps or a foot bath.	*	*
10. I know what complaints I need to pay attention to when it’s particularly hot.	0.617	*
11. When I'm on the road on sunny days at noon (eg visiting my doctor or going shopping), I usually stay in the shade or use air-conditioned means of transport.	*	0.459
12. I know which medications I need to adjust in case of heat.	0.702	*

***Factor loadings < 0.3 omitted; n: number of participants.**

Figure S3: Histograms of continuous independent variables (n=61)

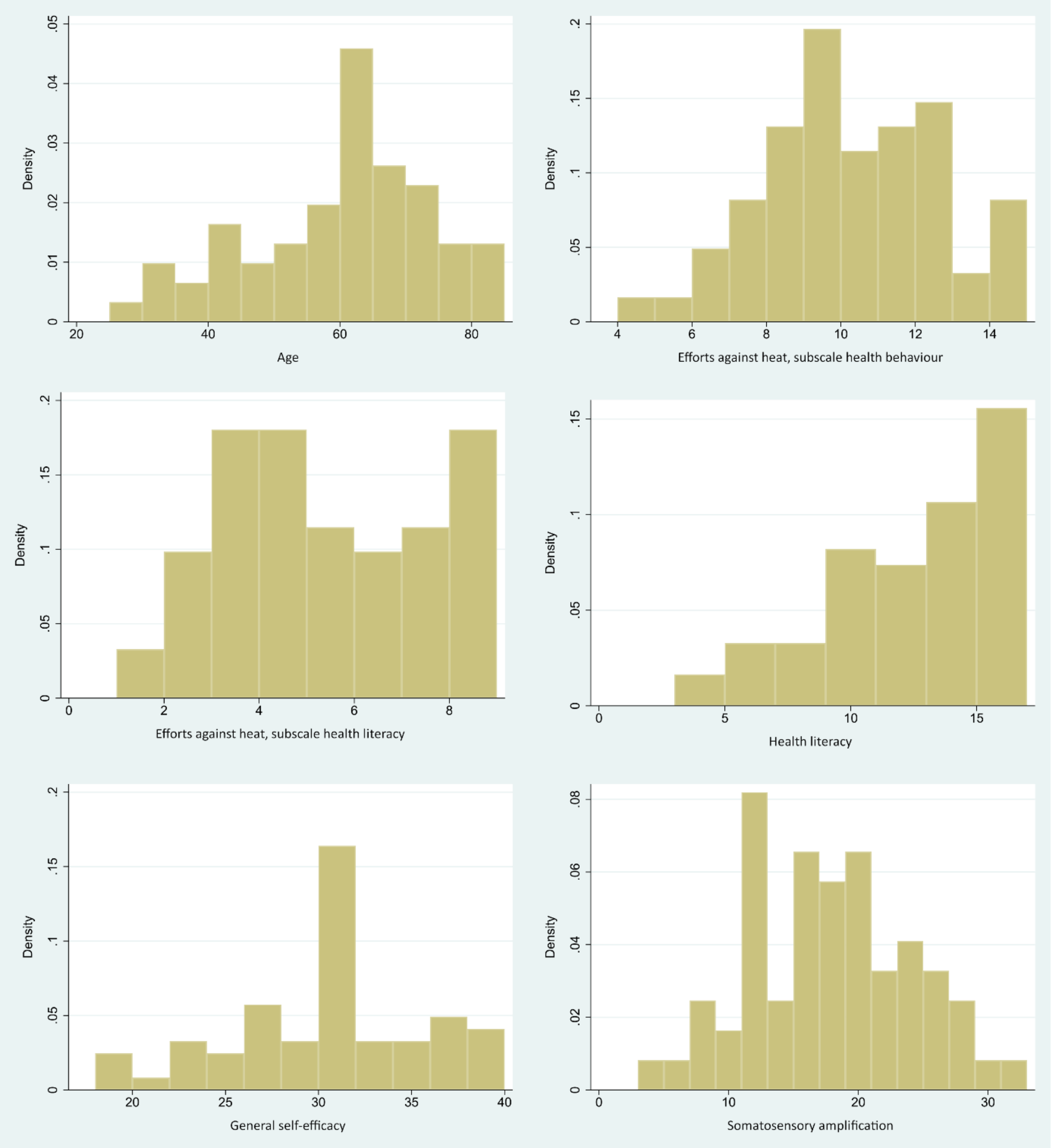

n: number of participants.

Table S2: Correlation matrix of independent variables (n=61)

	Age	Sex	Living alone	Education: secondary	Education: tertiary	Migration: at least one parent	Migration: participant	Disease: cardiovascular	Disease: diabetes mellitus	Disease: respiratory	Disease: renal insufficiency	Disease: psychiatric	Disease: neuropathy	Efforts: health behaviour	Efforts: health literacy	HLS-EU-Q16 health literacy	General self-efficacy	Somatosensory amplification
**Age**	1.00																	
**Sex**	-0.29	1.00																
**Living alone**	-0.02	0.20	1.00															
**Education: secondary**	-0.18	0.25	0.03	1.00														
**Education: tertiary**	0.10	-0.22	-0.09	***-0.74***	1.00													
**Migration: at least one parent**	-0.02	-0.04	-0.13	0.09	-0.06	1.00												
**Migration: participant**	-0.04	-0.13	-0.15	-0.10	0.18	-0.11	1.00											
**Disease: cardiovascular**	***0.46***	-0.22	0.03	-0.01	0.04	-0.04	0.07	1.00										
**Disease: diabetes mellitus**	0.00	-0.06	-0.07	0.08	-0.16	-0.09	-0.19	-0.18	1.00									
**Disease: respiratory**	-0.17	-0.12	-0.07	0.01	0.04	0.17	0.22	-0.19	-0.24	1.00								
**Disease: renal insufficiency**	0.00	0.00	-0.11	0.02	-0.15	-0.08	-0.05	-0.14	0.07	-0.09	1.00							
**Disease: psychiatric**	-0.16	0.27	0.06	0.23	-0.28	0.19	-0.12	-0.25	-0.03	-0.09	-0.08	1.00						
**Disease: neuropathy**	0.28	0.14	0.16	-0.10	0.05	-0.11	-0.07	0.07	-0.04	-0.12	-0.05	-0.12	1.00					
**Efforts: health behaviour**	***0.35***	0.19	-0.14	0.05	-0.15	-0.12	0.02	0.09	0.07	-0.03	0.05	0.04	0.24	1.00				
**Efforts: health literacy**	-0.02	0.04	-0.19	0.09	-0.03	-0.01	-0.15	-0.05	0.13	-0.04	-0.08	-0.12	0.08	0.05	1.00			
**HLS-EU-Q16 health literacy**	0.18	-0.09	***-0.31***	0.04	0.18	0.07	0.23	0.14	-0.12	0.12	0.03	-0.11	-0.05	-0.04	0.10	1.00		
**General self-efficacy**	0.15	-0.25	-0.15	0.07	0.09	-0.11	0.09	0.29	0.06	0.00	-0.17	***-0.46***	-0.02	-0.19	0.15	***0.42***	1.00	
**Somatosensory amplification**	0.04	0.22	-0.08	-0.01	0.00	0.06	0.05	0.10	-0.06	-0.03	-0.06	0.08	0.06	***0.38***	0.22	-0.08	-0.18	1.00

Correlation coefficients ≥ 0.30 are shown in bold and italic; n: number of participants.

Figure S4: Histogram of symptom burden score (n=61, N=294)

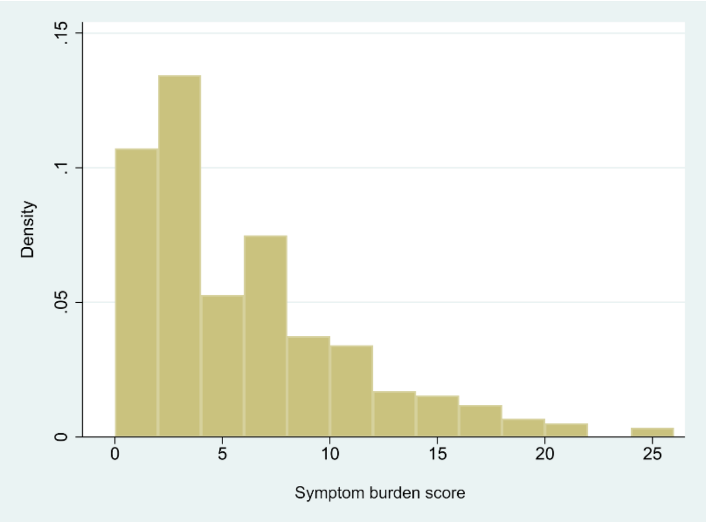

n: number of participants; N: number of observations.

Figure S5: Mean heat index and standard deviation by climate measuring station (n=61, N=294)

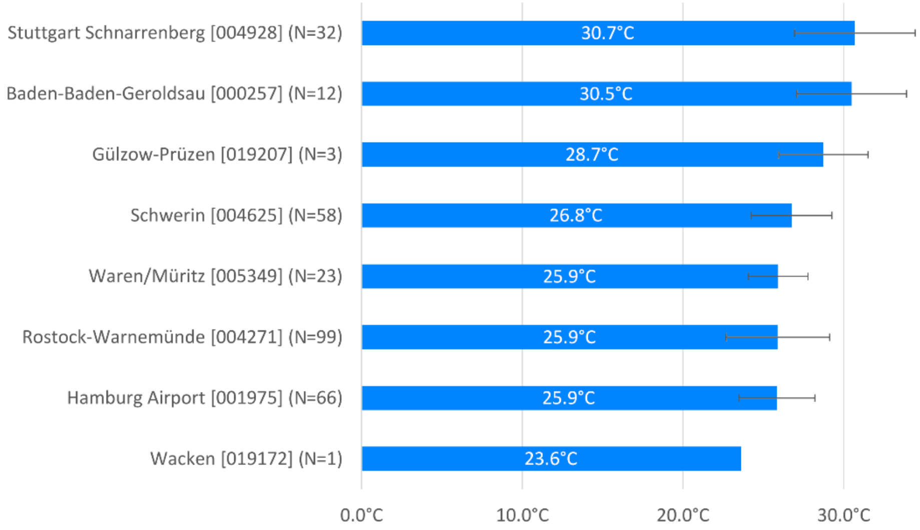

n: number of participants; N: number of observations.

Figure S6: Reported symptoms (n=61, N=294)

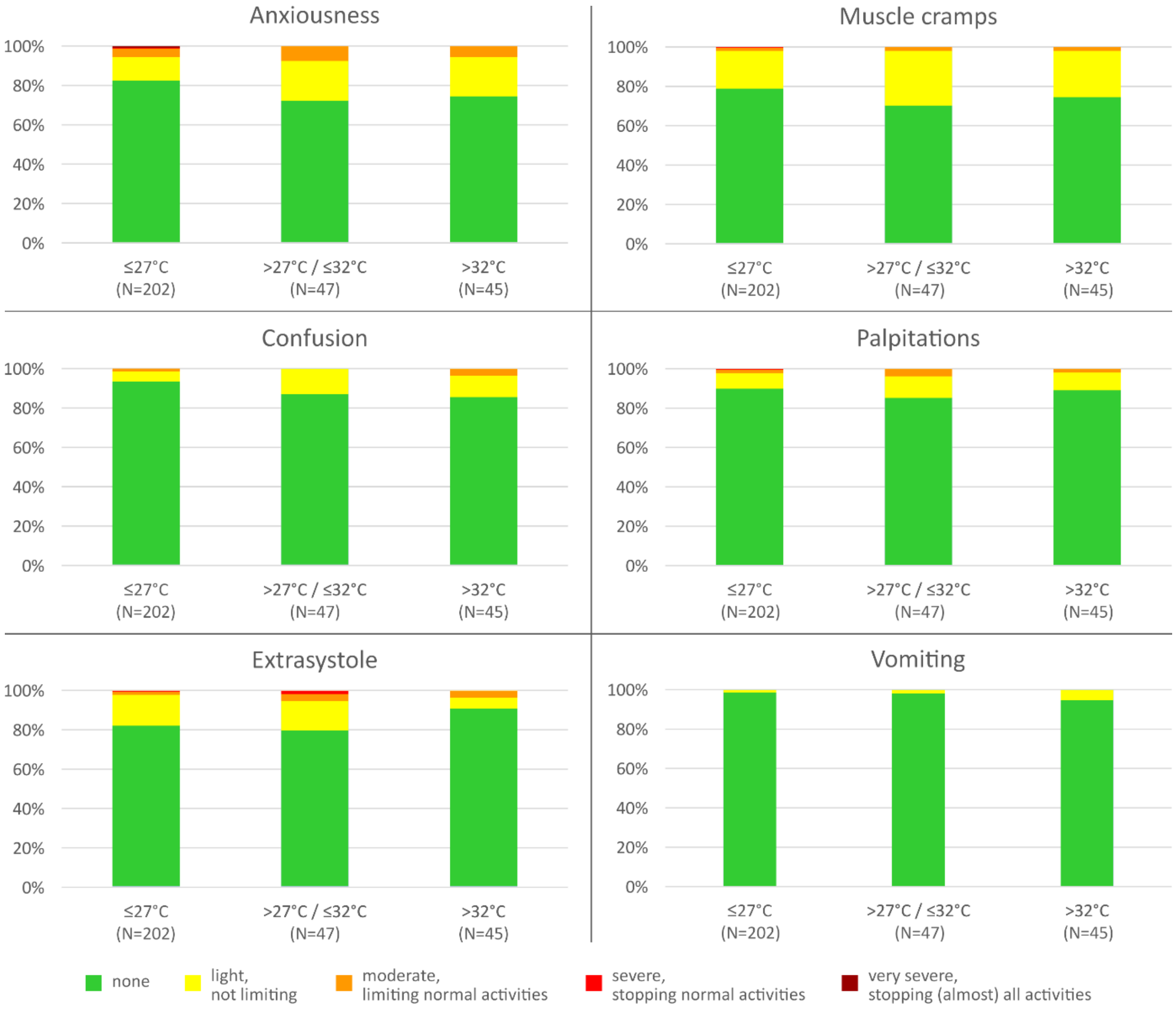

n: number of participants; N: number of observations.

Table S3: Means and standard deviations of the severity of reported symptoms by chronic disease clusters (n=61; N=294)

	Cardiovascular diseases (N=119)			Diabetes mellitus (N=100)			Respiratory diseases (N=53)			Psychiatric disorders (N=47)			Neuropathy (N=17)			Renal insufficiency (N=14)		
	yes	no	P	yes	no	p	yes	no	p	yes	no	p	yes	no	p	yes	no	p
**Summary score**	***3.52***	***6.71***	***<0.001***	***4.03***	***6.14***	***0.001***	5.40	5.43	0.967	***8.06***	***4.92***	***<0.001***	5.47	5.42	0.967	***10.71***	***5.16***	***<0.001***
**Tiredness/Fatigue**	***0.88***	***1.40***	***<0.001***	***1.01***	***1.28***	***0.011***	1.09	1.21	0.381	***1.68***	***1.10***	***<0.001***	1.24	1.19	0.829	1.50	1.18	0.178
**Depressiveness**	***0.33***	***0.91***	***<0.001***	***0.53***	***0.75***	***0.036***	0.87	0.63	0.075	***1.28***	***0.57***	***<0.001***	0.41	0.69	0.193	***1.21***	***0.65***	***0.017***
**Shortness of breath**	***0.56***	***0.82***	***0.014***	***0.48***	***0.84***	***0.001***	***1.09***	***0.63***	***<0.001***	0.79	0.70	0.534	0.41	0.73	0.142	***1.64***	***0.67***	***<0.001***
**Dizziness**	***0.19***	***0.48***	***<0.001***	0.28	0.41	0.083	0.23	0.39	0.063	***0.64***	***0.31***	***0.001***	0.53	0.35	0.239	0.64	0.35	0.073
**Circulatory problems /loss of consciousness**	***0.16***	***0.46***	***<0.001***	***0.21***	***0.41***	***0.006***	0.26	0.36	0.301	0.38	0.33	0.588	0.24	0.35	0.451	***0.71***	***0.32***	***0.015***
**Oedema (eg, in the legs)**	0.41	0.55	0.091	0.40	0.55	0.094	0.38	0.52	0.177	0.60	0.48	0.297	***0.94***	***0.47***	***0.008***	***1.36***	***0.45***	***<0.001***
**Headache**	***0.19***	***0.58***	***<0.001***	0.41	0.43	0.824	0.55	0.39	0.121	0.53	0.40	0.205	0.18	0.44	0.109	***1.57***	***0.36***	***<0.001***
**Nausea**	***0.12***	***0.26***	***0.012***	0.13	0.24	0.062	0.19	0.21	0.800	***0.47***	***0.15***	***<0.001***	0.12	0.21	0.453	***0.50***	***0.19***	***0.020***
**Anxiousness**	***0.08***	***0.42***	***<0.001***	***0.12***	***0.36***	***0.002***	0.25	0.29	0.642	***0.85***	***0.17***	***<0.001***	0.35	0.28	0.639	0.50	0.27	0.192
**Muscle cramps**	0.23	0.27	0.472	0.22	0.27	0.424	0.28	0.24	0.606	0.21	0.26	0.550	0.47	0.24	0.056	0.21	0.25	0.769
**Confusion**	***0.14***	***0.05***	***0.030***	0.06	0.13	0.119	0.09	0.11	0.804	***0.38***	***0.05***	***<0.001***	-	0.11	0.213	0.29	0.10	0.054
**Palpitations**	0.25	0.18	0.250	***0.09***	***0.27***	***0.003***	***0.04***	***0.25***	***0.006***	0.13	0.23	0.219	0.29	0.21	0.485	0.36	0.20	0.268
**Extrasystole**	***0.06***	***0.20***	***<0.001***	0.09	0.17	<0.136	0.08	0.16	0.216	0.09	0.15	0.324	0.29	0.13	0.142	0.21	0.14	0.532
**Vomiting**	-	***0.03***	***0.041***	-	0.03	0.076	-	0.02	0.247	***0.09***	***0.01***	***0.001***	-	0.02	0.541	-	0.02	0.582

Statistically significant results (p≤0.05) are shown in bold and italic; n: number of participants; N: number of observations.

Table S4: Association of symptom burden with heat and age: results of multivariable linear regression adjusted for random effects on the levels of climate measuring stations and patients (n=61, N=294).

	ß (95% CI)	P
Heat index risk level:		
- 27°C or less	***reference***	
- more than 27°C through 32°C	***0.98 (0.04/1.93)***	***0.041***
- more than 32°C	***1.37 (0.37/2.38)***	***0.007***
Age	-0.02 (-0.09/0.06)	0.689

Statistically significant results (p≤0.05) are shown in bold and italic; 95% CI: 95% confidence interval; n: number of participants; N: number of observations.

Table S5: Association of symptom burden with heat and sex: results of multivariable linear regression adjusted for random effects on the levels of climate measuring stations and patients (n=61, N=294).

	ß (95% CI)	p
Heat index risk level:		
- 27°C or less	***reference***	
- more than 27°C through 32°C	***0.99 (0.04/1.93)***	***0.041***
- more than 32°C	***1.37 (0.37/2.38)***	***0.007***
Sex		
- men	***reference***	
- women	***2.16 (0.14/4.18)***	***0.036***

Statistically significant results (p≤0.05) are shown in bold and italic; 95% CI: 95% confidence interval; n: number of participants; N: number of observations.

Table S6: Association of symptom burden with heat and living arrangement: results of multivariable linear regression adjusted for random effects on the levels of climate measuring stations and patients (n=61, N=294).

	ß (95% CI)	p
Heat index risk level:		
- 27°C or less	***reference***	
- more than 27°C through 32°C	***0.99 (0.04/1.93)***	***0.041***
- more than 32°C	***1.38 (0.38/2.39)***	***0.007***
Living arrangement		
- living together with others	reference	
- living alone	0.26 (-2.21/2.73)	0.836

Statistically significant results (p≤0.05) are shown in bold and italic; 95% CI: 95% confidence interval; n: number of participants; N: number of observations.

Table S7: Association of symptom burden with heat and educational level: results of multivariable linear regression adjusted for random effects on the levels of climate measuring stations and patients (n=61, N=294).

	ß (95% CI)	p
Heat index risk level:		
- 27°C or less	***reference***	
- more than 27°C through 32°C	***1.02 (0.08/1.97)***	***0.034***
- more than 32°C	***1.42 (0.42/2.42)***	***0.005***
Educational level		
- tertiary	***reference***	
- secondary	1.92 (-0.21/4.05)	0.129
- primary or below	***4.19 (1.23/7.16)***	***0.006***

Statistically significant results ($p \leq 0.05$) are shown in bold and italic; 95% CI: 95% confidence interval; n: number of participants; N: number of observations.

Table S8: Association of symptom burden with heat and country of birth: results of multivariable linear regression adjusted for random effects on the levels of climate measuring stations and patients (n=61, N=294).

	ß (95% CI)	p
Heat index risk level:		
- 27°C or less	***reference***	
- more than 27°C through 32°C	***0.98 (0.03/1.92)***	***0.042***
- more than 32°C	***1.34 (0.33/2.35)***	***0.009***
Country of birth		
- participant and parents born in Germany	reference	
- participant born in Germany, at least one parent abroad	1.33 (-1.60/4.25)	0.374
- participant born abroad	-1.59 (-5.86/2.68)	0.464

Statistically significant results ($p \leq 0.05$) are shown in bold and italic; 95% CI: 95% confidence interval; n: number of participants; N: number of observations.

Table S9: **Association of symptom burden with heat and chronic diseases: results of multivariable linear regression adjusted for random effects on the levels of climate measuring stations and patients (n=61, N=294).**

	ß (95% CI)	p
Heat index risk level:		
- 27°C or less	***reference***	
- more than 27°C through 32°C	***0.99 (0.05/1.93)***	***0.040***
- more than 32°C	***1.35 (0.35/2.35)***	***0.008***
Chronic diseases		
- cardiovascular diseases	-2.02 (-4.06/0.01)	0.051
- diabetes mellitus	***-2.61 (-4.61/-0.60)***	***0.011***
- respiratory diseases	-0.41 (-2.93/2.12)	0.753
- psychiatric disorders	1.42 (-2.22/5.07)	0.443
- neuropathy	***3.01 (0.45/5.57)***	***0.021***
- renal insufficiency	***5.85 (0.90/10.80)***	***0.021***

Statistically significant results (p≤0.05) are shown in bold and italic; 95% CI: 95% confidence interval; n: number of participants; N: number of observations.

Table S10: **Association of symptom burden with heat and efforts against adverse effects of heat: results of multivariable linear regression adjusted for random effects on the levels of climate measuring stations and patients (n=61, N=294).**

	ß (95% CI)	p
Heat index risk level:		
- 27°C or less	***reference***	
- more than 27°C through 32°C	***1.03 (0.08/1.97)***	***0.033***
- more than 32°C	***1.46 (0.45/2.46)***	***0.004***
Efforts against adverse effects of heat		
- subscale health behaviour	***0.64 (0.24/1.04)***	***0.002***
- subscale health literacy	-0.16 (-0.58/0.26)	0.447

Statistically significant results (p≤0.05) are shown in bold and italic; 95% CI: 95% confidence interval; n: number of participants; N: number of observations.

Table S11: **Association of symptom burden with heat and health literacy: results of multivariable linear regression adjusted for random effects on the levels of climate measuring stations and patients (n=61, N=294).**

	ß (95% CI)	p
Heat index risk level:		
- 27°C or less	***reference***	
- more than 27°C through 32°C	***1.01 (0.06/1.95)***	***0.036***
- more than 32°C	***1.42 (0.42/2.42)***	***0.006***
Health literacy	***-0.34 (-0.63/-0.05)***	***0.021***

Statistically significant results (p≤0.05) are shown in bold and italic; 95% CI: 95% confidence interval; n: number of participants; N: number of observations.

Table S12: **Association of symptom burden with heat and general self-efficacy: results of multivariable linear regression adjusted for random effects on the levels of climate measuring stations and patients (n=61, N=294).**

	ß (95% CI)	p
Heat index risk level:		
- 27°C or less	***reference***	
- more than 27°C through 32°C	0.92 (-0.01/1.86)	0.053
- more than 32°C	***1.33 (0.33/2.32)***	***0.009***
General self-efficacy	***-0.52 (-0.67/-0.37)***	***<0.001***

Statistically significant results (p≤0.05) are shown in bold and italic; 95% CI: 95% confidence interval; n: number of participants; N: number of observations.

Table S13: **Association of symptom burden with heat and somatosensory amplification: results of multivariable linear regression adjusted for random effects on the levels of climate measuring stations and patients (n=61, N=294).**

	ß (95% CI)	p
Heat index risk level:		
- 27°C or less	***reference***	
- more than 27°C through 32°C	***1.02 (0.08/1.96)***	***0.034***
- more than 32°C	***1.38 (0.37/2.38)***	***0.007***
Somatosensory amplification	***0.25 (0.09/0.41)***	***0.002***

Statistically significant results (p≤0.05) are shown in bold and italic; 95% CI: 95% confidence interval; n: number of participants; N: number of observations.
